# MXD3 Promotes Obesity and the Androgen Receptor Signaling Pathway in Gender-Disparity Hepatocarcinogenesis

**DOI:** 10.3390/cells10123434

**Published:** 2021-12-06

**Authors:** Yi-Wen Tsai, Kuo-Shyang Jeng, Mu-Kuang He, Yang-Wen Hsieh, Hsin-Hung Lai, Chi-Yu Lai, Chun-Chieh Huang, Chiung-Fang Chang, Chung-Tsui Huang, Guor Mour Her

**Affiliations:** 1 Department of Family Medicine, Chang Gung Memorial Hospital, Keelung 204, Taiwan; tsaiyiwen@gmail.com; 2 College of Medicine, Chang-Gung University, Taoyuan 333, Taiwan; 3 Division of General Surgery, Far Eastern Memorial Hospital, New Taipei 220, Taiwan; kevin.ksjeng@gmail.com (K.-S.J.); changcf@femh.org.tw (C.-F.C.); 4 Taipei First Girls High School, Taipei 100, Taiwan; d10830808@gapps.fg.tp.edu.tw; 5 Department of Bioscience and Biotechnology, National Taiwan Ocean University, Keelung 202, Taiwan; hearhero@hotmail.com; 6 Institute of Biopharmaceutical Sciences, National Yang Ming Chiao Tung University, Taipei 112, Taiwan; s232579@gmail.com (H.-H.L.); c.y.stephen.lai@gmail.com (C.-Y.L.); 7 Department of Radiology, Far Eastern Memorial Hospital, New Taipei 220, Taiwan; cc.huang0114@gmail.com; 8 Department of Internal Medicine, Division of Gastroenterology and Hepatology, Far Eastern Memorial Hospital, New Taipei 220, Taiwan; 950286169@mail.femh.org.tw

**Keywords:** obesity, hepatic steatosis, steatohepatitis, fibrogenesis, liver cancer

## Abstract

Obesity is closely linked to metabolic diseases, particularly non-alcoholic steatohepatitis (NASH) or non-alcoholic fatty liver disease (NAFLD), ultimately leading to hepatocellular carcinoma (HCC). However, the molecular mechanisms of NASH-associated HCC (NAHCC) remain elusive. To explore the impact of Max dimerization protein 3 (MXD3), a transcription factor that regulates several cellular functions in disorders associated with metabolic diseases, we conditionally expressed Mxd3 proteins using Tet-on *mxd3* transgenic zebrafish (MXs) with doxycycline (MXs + Dox) or without doxycycline (MXs − Dox) treatment. Overexpression of global MXD3 (gMX) or hepatic Mxd3 (hMX) was associated with obesity-related NAFLD pathophysiology in gMX + Dox, and liver fibrosis and HCC in hMX + Dox. Oil Red O (ORO)-stained signals were seen in intravascular blood vessels and liver buds of larval gMX + Dox, indicating that Mxd3 functionally promotes lipogenesis. The gMX + Dox-treated young adults exhibited an increase in body weight and visceral fat accumulation. The hMX + Dox-treated young adults showed normal body characteristics but exhibited liver steatosis and NASH-like phenotypes. Subsequently, steatohepatitis, liver fibrosis, and NAHCC were found in 6-month-old gMX + Dox adults compared with gMX − Dox adults at the same stage. Overexpression of Mxd3 also enhanced AR expression accompanied by the increase of AR-signaling pathways resulting in hepatocarcinogenesis in males. Our results demonstrate that global actions of Mxd3 are central to the initiation of obesity in the gMX zebrafish through their effects on adipogenesis and that MXD3 could serve as a therapeutic target for obesity-associated liver diseases.

## 1. Introduction

MAX dimerization protein 3 (MXD3) is a basic helix–loop–helix transcription factor associated with the MYC/MAX/MAD transcriptional network [1]. The MYC/MAX/MAD network was shown to be important for the regulation of cell proliferation, differentiation, and apoptosis [2]. MXD3 is a unique MAD family member protein, which competes with MYC for heterodimerization with the cofactor MAX, and is a functional MYC antagonist [1,3]. MYC has been shown to promote proliferation in several cell types, and the MAD proteins function as a “transcriptional repressor” to strongly antagonize MYC activity, resulting in inhibition of cellular proliferation and transformation [3,4,5,6]. Other members of the MXD family (MXD1, MXI1, and MXD4) are abundantly present in post-mitotic cells, while MXD3 is discovered in mitotic cells during the S-phase of the cell cycle [7,8].

MXD3 deficiency resulted in an enlarged sensitivity to apoptosis in return for DNA impairment [9]; however, MXD3 transcripts and proteins have also been recognized in proliferating cells [5,7]. These findings suggest that MXD3 proteins may function not only in differentiation, but also in processes involved in other aspects of cellular growth control. Aberrant expression levels of MXD3 have been seen in numerous cancers. Previously, MXD3 overexpression was detected to elevate in glioblastoma [10], medulloblastoma [11], acute lymphoblastic leukemia cell proliferation [12], renal cell carcinoma [13], and hepatocellular carcinoma (HCC) [14]. These varying results relate to the functions of MXD3 imply tissue-specific or pathophysiological roles of MXD3, depending on different tissues and developmental stages.

Recent studies have also demonstrated that prolonged MXD3 expression could increase lipogenesis and adipogenesis and have an effect on oxidative stress [15], which also drive lipid deposition and boost obesity in zebrafish [16]. Depletion of *mxd3* conquers visceral fat in diet-induced obese (DIO) zebrafish and Mxd3 ameliorates adipocyte differentiation in the DIO zebrafish. A molecular analysis revealed that the expression of adipogenic transcriptional factors, including peroxisome proliferator activated receptor gamma (*ppar-γ)***,** C/EBP-*α*/*β*/*γ*, caveolin 1 (*cav1*), and collagen domain containing b (*adipoqb*) decrease by *mxd3* depletion in 3T3-L1 cells and DIO zebrafish [17]. Obesity is defined as unusual fat addition in adipose tissue, and the liver closely communicates with adipose tissue [18,19]. MXD3 appears to be linked with various human cancers and adipogenesis. Several large-scale clinical studies have shown a connection between individuals with “plain” obesity and a larger risk of HCC, when compared to “non-obese” people [20,21,22]. MXD3 is also suggested as a diagnostic biomarker for HCC [14]. Considering these data, the role of MXD3 in liver cancers is interesting, and its role in obesity and HCC is worth investigating.

Zebrafish has recently become a significant model organism for studying obesity-related human diseases [16,23,24,25,26,27,28]. The aim of this study was to discover the Mxd3 relative to signaling pathways, which influence metabolic physiology in zebrafish models. We produced Mxd3 transgenic zebrafish to analyze the functional roles of the zebrafish Mxd3 protein during development. Here, we present evidence that Mxd3 promotes an early onset of adipocyte proliferation. Moreover, these findings shed light on the pivotal role of Mxd3 in obesity-related HCC, and could serve as a guide for further preclinical research.

## 2. Materials and Methods

### 2.1. Generation of Mxd3 Transgenic Zebrafish

The transgenic zebrafish strains, gMX (*Tg(-2.53β-Act:Tet^on^-2A-ZsGreen-mxd3-2A-mCherry)*), which shows global expression of mCherry and has *mxd3* driven by the β-actin promoter [16,27,29,30], and hMX (*Tg(-2.8fabp10a:Tet^on^2A-ZsGreen-mxd3-2A-mCherry)*), which shows inducible liver-specific *mxd3* expression under the control of *fabp10a* promoter [30,31,32,33,34], were raised at 28 °C on a 14 h:10 h light/dark cycle. All lines were maintained in compliance with the Institutional Animal Care and Use Committee (IACUC) guidelines.

### 2.2. Treatment with Doxycycline (Dox)

Zebrafish embryos and juvenile adults were treated by immersion in Dox at 25 μg/mL (Selleck Chemicals, Houston, TX, USA) in six-well plates and 3-L tanks, individually, and the water was exchanged daily.

### 2.3. Whole-Mount Oil Red O (ORO) Staining

Whole-mount Oil Red O (ORO) staining was performed as described previously [16,27,29,30,33,34,35].

### 2.4. Alamar Blue Metabolic Activity Assay

The datasets of metabolic activity assays were conducted as previously described [36].

### 2.5. Blood Analysis

Blood was collected, as reported by previously published research by Renquist [37]. The collected blood samples were pooled and used for quantification of leptin (Fish Leptin ELISA Kit, MBS021271, MyBioSource, San Diego, CA, USA) and adiponectin (Fish Adiponectin ELISA Kit, MBS098337, MyBioSource San Diego, CA, USA) by ELISA, and followed the manufacturer’s instructions. Data were expressed as percent variances (Δ%) of fish weight gain that occurred during 8 weeks of normal feeding.

### 2.6. Quantitative Reverse Transcription PCR (qRT-PCR)

Total RNA was extracted from zebrafish using EasyPure Total RNA Reagent (Bioman) and column-purified with EasyPure Total RNA Spin Kit Tissue (Bioman) afterwards. For qRT-PCR, 100 ng of total RNA was reverse transcribed using PrimeScript RT Reagent Kit (TaKaRa, Beijing, China). The expression of all selected genes was performed using CFX Connect System (Bio-Rad, Hercules, CA, USA) with SYBR Premix EX Taq^TM^ II (TaKaRa). The GenBank accession numbers for the selected genes and primer sequences were previously reported [16,27,28,29,30,33,34,35].

### 2.7. Growth Rate

Growth rate studies were performed,, as described previously [16,28]. The growth curve was measured monthly from 2 months post fertilization (mpf) to 12 mpf. All groups containing 25 fish (mixed sex) were picked randomly. Body weights (BW) were measured to 0.01 g monthly, a standard metric ruler was used to measured body lengths (BL) to 1 mm, and the length from the tip of the snout to the caudal peduncle was recorded. Body weights and body lengths of anesthetized juveniles and adults were recorded for the calculation of the body mass index (BMI).

### 2.8. Biochemical Analysis of Hepatic Lipid in Zebrafish

The biochemical analyses were performed as described previously [16,27,29,30,33,34,35].

### 2.9. Morphometric Studies: Anatomy of Zebrafish Adipose Tissues

These methods were performed as described previously [16,28,37].

### 2.10. Western Blot Analysis

The protein lysate samples from zebrafish liver tissue were measured by western blotting, as described previously [30,31]. The incubation of PVDF membrane was in antibodies to the following antibodies: anti-MXD3 (1:1500, ab108525, Abcam, Cambridge, UK), anti-androgen receptor (1:1000, ab74272, Abcam, Cambridge, UK), anti-p-STAT3 (1:1000, D128-3, MBL International, Woburn, MA, USA), anti-TGF-β1 (1:1500, sc-130348, Santa Cruz Biotechnology Inc., Santa Cruz, CA, USA), anti-CCRK (1:2000, ab227077, Abcam, Cambridge, UK), anti-GRP78 (1:1500, MAB4846, R&D Systems), anti-VEGFA (1:1500, ab51745, Abcam) and GAPDH (1:9000, no. 2118L, Cell Signaling, Danvers, MA, USA). The secondary antibody used was IgG—horseradish peroxidase (HRP) (1:5000, AB_10015289 or AB_2313567, Jackson ImmunoResearch Labs, West Grove, PA, USA).

### 2.11. Histology and Immunohistochemistry

Immunohistological analyses were performed on the section of paraffin-embedded tissues. The histological sections were incubated in the following antibodies: MXD3 (1:250, ab108525, Abcam), androgen receptor (1:250, ab74272, Abcam, Cambridge, UK), and PCNA (1:1500, sc-7907, Santa Cruz Biotechnology Inc., Santa Cruz, CA, USA). Hematoxylin and Eosin (H&E) was performed and Masson’s trichrome staining was carried out using Masson’s Trichrome Stain kit (Polysciences, Inc., Warrington, PA, USA).

### 2.12. Sex Hormone Treatment

These experiments were performed on histological sections according to a method described by Li et al. [38,39]. For sex hormone treatment, 5 μg/mL 17β-estradiol (E2) (E4389, Sigma-Aldrich, St. Louis, MO, USA) or 11-ketotestosterone (11-KT) (Steraloids, Newport, RI, USA) was used for four mpf zebrafish. During Dox induction, E2 or 11-KT was used to treat zebrafish for 2 weeks. After removing Dox, male zebrafish were treated by E2 or 11-KT for 1 week. Histological analysis was accomplished in the first, third, and ninth month post-treatment.

### 2.13. Statistical Analysis

All data are represented as the mean ± SEM. GraphPad Prism 8.0 software (GraphPad, San Diego, CA, USA) was used for statistical analyses. A *p*-value of < 0.05 was considered statistically significant.

## 3. Results

### 3.1. Production of Transgenic mxd3 Zebrafish Lines (MXs) 

**Figure 1:**
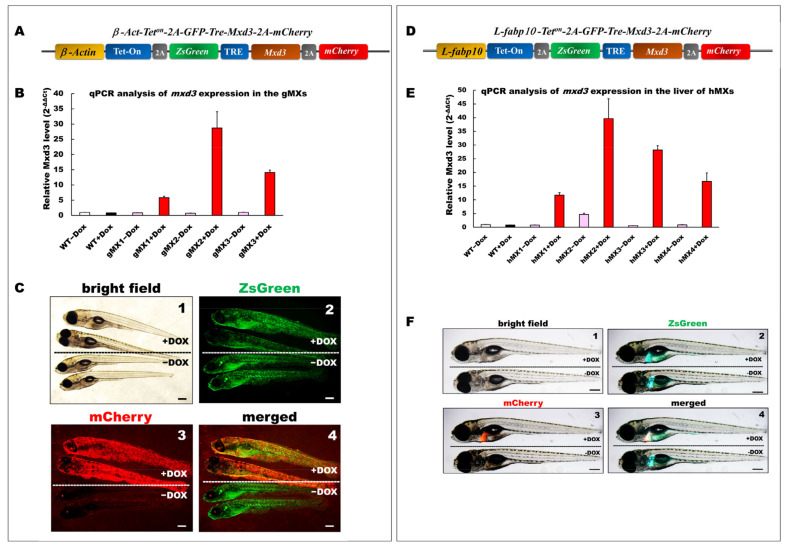
Generation and depiction of inducible Mxd3 transgenic zebrafish lines, gMXs and hMXs. (**A**) Schematic drawing of the DNA construct used to produce gMX (*Tg(-2.5β-Act:Tet^on^-2A-ZsGreen-mxd3-2A-mCherry*) transgenic zebrafish. (**B**) Quantification of *mxd3* expression using (qRT-PCR) analysis. gMX1-3 treated with doxycycline (+Dox) and without (−Dox) represent three discrete transgenic lines. Control: wild type (WT)+Dox and WT−Dox. (**C**) Globally inducible Mxd3 expression in gMX2 at 6 days post fertilization (dpf). Transgenic larvae were treated with 25 μg/mL Dox from 24 to 6 dpf. Scale bar: 100 μm. (**D**) Schematic drawing of the plasmid construct used to produce hMX(*Tg(-2.8fabp10a:Tet^on^-2A-ZsGreen-mxd3-2A-mCherry)*) transgenic zebrafish. (**E**) Quantification of *mxd3* expression using (qRT-PCR) analysis. hMX1-4 treated with doxycycline (+Dox) and without (−Dox) represent four discrete transgenic lines. Control: WT+Dox and WT−Dox. (**F**) Liver-specific inducible Mxd3 expression in the hMX3 at 7 dpf. Transgenic larvae were treated with 25 μg/mL Dox from 2 to 7 dpf. Scale bar: 100 μm.

We detected varying extents of endogenous *mxd3* mRNA in several tissues of adult zebrafish, including the intestine, liver, muscle, adipose tissue, eye, and brain (data not shown). To generate an inducible expression of *mxd3* in zebrafish and its liver, pβAct-Tet^on^-2A-ZsGreen-mxd3-2A-mCherry and pLF2.8-Tet^on^-2A-ZsGreen-mxd3-2A-mCherry plasmids were used to generate stable transgenic zebrafish lines (Figure 1A,D). The gMX (*Tg(-2.5β-Act:Tet^on^-2A-ZsGreen-mxd3-2A-mCherry)*) transgenic lines were generated, in which the Mxd3 was globally expressed (Figure 1B). In addition, hMX (*Tg(-2.8fabp10a:Tet^on^-2A-ZsGreen-mxd3-2A-mCherry)*) lines were generated, in which the *mxd3* was particularly expressed in the liver (Figure 1E). 

Three gMX (gMX1, gMX2, and gMX3) and four hMX transgenic lines (hMX1, hMX2, hMX3, and hMX4) were selected on the basis of their *mxd3* expression using qRT-PCR (Figure 1B,E). Compared with that in the wild type (WT), *mxd3* was significantly overexpressed, 5.8-, 28.7-, and 14.1-fold in gMX1, gMX2, and gMX3 with doxycycline treatment (+Dox), respectively, and no *mxd3* was detected in control groups without Dox (−Dox). gMX2 displayed no noticeable phenotypic variations in other lines at larval stages compare to WT ± Dox (Figure 1C, panel 1); GFP fluorescence was seen in gMXs ± Dox larvae (Figure 1C, panel 2). On the other hand, no mCherry fluorescence was seen in gMX2–Dox larvae (Figure 1C, panel3), whereas the strong global mCherry as well as GFP fluorescence was present in gMX2+Dox larvae (Figure 1C, panel 4). For hMXs, 11.7-, 39.6-, 28.2-, and 9.8-fold *mxd3* expression were detected in the livers of hMX1, hMX2, hMX3, and hMX4, respectively, compared with that of WT controls. However, there was slight leakage (−Dox treatment for 4.7-fold expression) in the hMX2. hMX3 displayed no noticeable phenotypic variations in other founders at larval stages compare to WT (Figure 1F, panel 1); only GFP fluorescence was observed in the livers of hMX3−Dox larvae (Figure 1F, panel 2). The clear mCherry and GFP fluorescence was observed in hMX3 + Dox larvae, but no mCherry fluorescence was seen in hMX3 − Dox larvae (Figure 1F, panels 3 and 4). These results suggest that the gMX2 and hMX3 lines expressed the highest levels of *mxd3* expression without leakage in induction. Thus, in the present study, a majority of experiments were performed using gMX2 and hMX3 zebrafish.

### 3.2. Mxd3 Overexpression Increases the Early Onset of Adipogenesis in gMX and the Liver Steatosis in hMX Larvae and Juveniles

**Figure 2:**
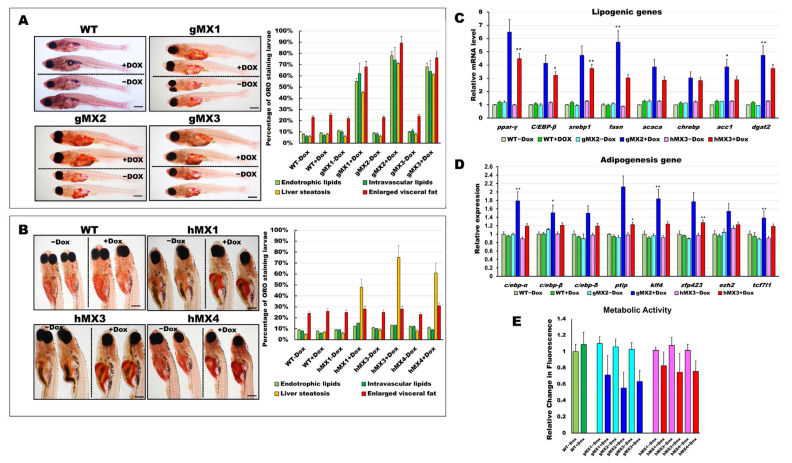
Characterization of early onset of adipogenesis and energy balance in gMXs and hMXs larvae. (**A**) Left: whole-mount ORO staining of WT ± Dox and gMXs (1–3) ± Dox larvae at 24 dpf. Scale bar: 100 μm. Right: the percentage of ORO-stained zebrafish larvae. (**B**) Left: whole-mount ORO staining of WT ± Dox and hMXs (1, 3, and 4) ± Dox larvae at 24 dpf. Scale bar: 125 μm. Right: the percentage of ORO-stained zebrafish larvae. (**C**) Molecular analysis of gMX2 and hM3 larvae revealed the upregulation of lipogenic genes, *ppar-γ, C/EBP-β, srebp1, fasn, acaca, chrebp, acc1,* and *dgat2*. (**D**) Molecular analysis of gMX2 and hM3 larvae revealed an upregulation of adipogenesis genes, *c/ebp-α, c/ebp-β, c/ebp-δ, ptip, klf4, zfp423, ezh2, and tcf7l1*. (**E**) Response to gMX2 and hM3 larvae by the Alamar Blue assay. Data are conveyed as the relative difference in signal intensity in three discrete assays (mean ± SEM, *n* = 40), Statistically significant differences from the controls are indicated by * (*p* < 0.05), and ** (*p* < 0.01).

To examine neutral lipids among MXs (hMXs and gMXs), 24 dpf larvae were stained with ORO (Figure 2). The gMXs + Dox larvae showed strong staining in early visceral fat pads and in the liver and brain, and moderate staining in the heart with additional staining in the vasculature compared with control groups (WT and gMX − Dox) (Figure 2A, right). Significantly, the incidence of endotrophic (55−78%), intravascular (62−74%) lipid accumulation, liver steatosis (45–71%), and visceral fat pads (68−89%) was much higher in gMXs compared with overall lipid accumulation (< 9%) and < 25% visceral fat pads in control larvae (WT ± Dox and gMXs − Dox) (Figure 2A, left). The hMXs + Dox larvae showed clear ORO staining only in the liver (Figure 2B, right). Markedly, the occurrence of lipid deposits was significantly higher in the liver of hMXs + Dox larvae (48–75%) than that in WT larvae (< 7%) and hMXs − Dox (< 10%) (Figure 2B, left).

We assumed that an early onset of adipogenesis occurred in the gMXs larvae and NAFLD phenotypes in the hMXs larvae. Molecular analysis of the gMXs larvae showed upregulated genes involved in both lipogenesis and adipogenesis; however, upregulation of only the lipogenic genes was observed in hMXs larvae (Figure 2C,D). Furthermore, to evaluate the probable roles of Mxd3 in the metabolism of gMXs or hMXs in further examinations on energy expenses that might influence obesity, we carried out the Alamar Blue metabolic assay in 6 dpf larvae. gMXs+Dox larvae displayed significantly lower metabolic rates (56–72%), and hMXs exhibited metabolic rates of 68–84% compared with control groups (Figure 2E). The assay showed increasing signals of the MXs+Dox corresponding to incubation times compared with the controls, and the results confirmed that lipids were accumulated as an energy reserve, as reflected by the occurrence of hepatic steatosis and adipogenesis. These data indicate that *mxd3* overexpression can provoke lipid accumulation in zebrafish larvae.

### 3.3. Adult gMXs+Dox Are Overweight and Have Increased Adiposity

**Figure 3:**
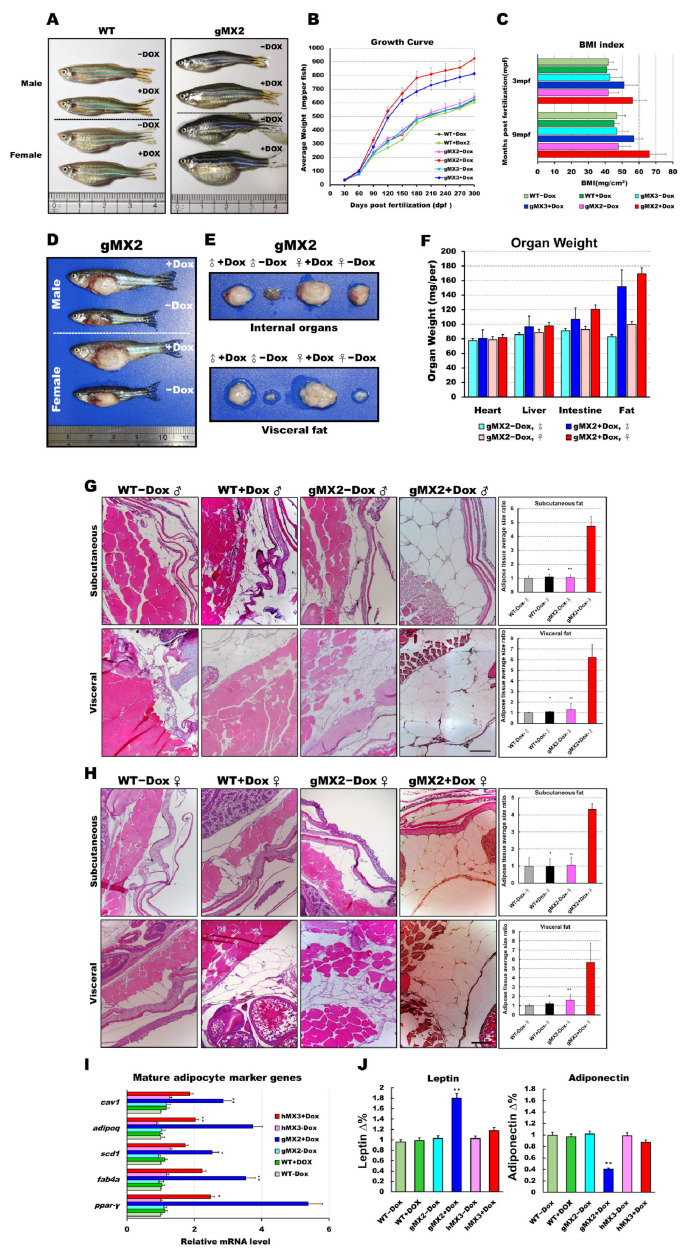
Expression levels of *mxd3* regulate somatic growth and obesity in gMX2 adults. (**A**) Macroscopic view of male and female gMX2 adults compared with WT controls at 10 mpf. (**B**) Growth curves of zebrafish from juvenile to adult stages in two discrete groups (WT ± Dox, gMX2 ± Dox, and hMX3 ± Dox; *n* = 24/group). (**C**) Bar chart displaying the BMI index in two discrete groups (*n* = 25/group) at 3 and 10 mpf stages. (**D**) Viscera and visceral fat pads of gMX2 groups at 12 mpf, displaying (**E**) enlarged internal organs, and (**F**) fat pad size. (**G**,**H**) Histological characteristics of adipose tissue in H&E-stained sections displaying (**G**) male and (**H**) female samples. Left: H&E-stained sections displaying adipose tissues of WT ± Dox and gMX2 ± Dox male groups (*n* = 6/group). Right: morphometric analysis of fat on adipose tissues of average size in each group (*n* = 6/group). (**I**) Molecular analysis of visceral adipose tissue of gMX2 and hMX3 groups revealed upregulation of mature adipocyte markers genes, such as *ppar-γ, fabp4, scd1, adipoq,* and *cav1*. (**J**) Blood analysis of leptin and adiponectin. Statistically significant differences from the controls are indicated by * (*p* < 0.05), and ** (*p* < 0.01).

To investigate whether overexpression of Mxd3 has a physiological effect on obesity, we inspected the appearance of fat tissues of gMX adults and revealed that they displayed a vivid “reply” to weight gain or obesity. gMXs + Dox adults were overweight and larger compared with WT (Figure 3A). After 9 months, we discovered that gMXs + Dox zebrafish showed a drastically keen response to the normal diet (Figure 3B). In line with the growth rate, the body mass index (BMI) of gMX2 + Dox and gMX3 + Dox adults was intensely amplified within the 9 months of feeding (Figure 3C). This intensification of weight gain corresponded with a manifest increase in the internal organs and visceral fat in the gMX2 + Dox adults (Figure 3D–F). We also noticed an enlarged cell mass of subcutaneous and visceral adipocytes (Figure 3G,H, left) in the gMX2 + Dox group; an increased percentage of gMX2 + Dox exhibited visceral adipocyte hyperplasia compared with the control groups (Figure 3G,H, right). qRT-PCR analyses showed that *mxd3* overexpression increased in mature adipocyte marker genes (Figure 3I). In addition, we examined whether Mxd3 modulates adipokines in the adipose tissue of the gMX2 group and primarily accompanies obesity effects. We observed that the adiposity of gMX2 also accompanied the upregulated expression of leptin and downregulated expression of adiponectin (Figure 3J). Thus, Mxd3 overexpression led to hyperplasia of adipose tissue in zebrafish, thereby involving an impact of Mxd3 on growth and obesity.

### 3.4. Adult hMXs+Dox Fish Develop Various Grades of NAFLD

**Figure 4:**
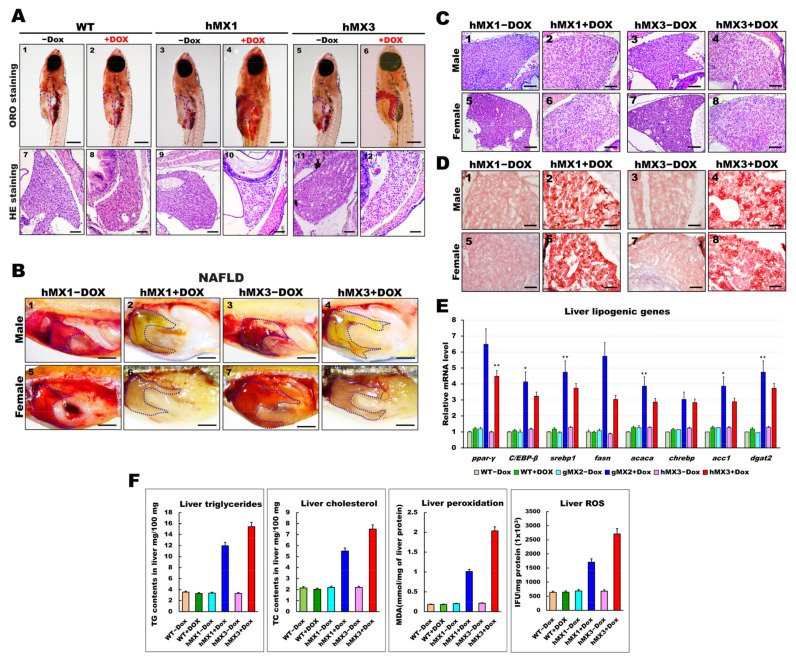
Development of NAFLD phenotypes in hMX1 and hMX3 juvenile (< 30 dpf) and adult zebrafish related to various fat accumulation. (**A**) ORO staining of WT ± Dox (panels 1 and 2), hMX1±Dox (panels 3 and 4), and hMX3 ± Dox (panels 5 and 6) juvenile zebrafish at 24 dpf (scale bars: 200 m). The liver regions are circled. H&E staining of liver sections of WT ± Dox (panels 7 and 8), hMX1 ± Dox (panels 9 and 10), and hMX3 ± Dox (panels 11 and 12) juvenile zebrafish at 24 dpf. Scale bars: 100 μm. (**B**) Gross liver images of hMX1 ± Dox and hMX3 ± Dox males (panels 1–4) and females (panels 5–8) at 4 mpf. Scale bar: 2 mm). (**C**) H&E staining of liver sections of hMX1 ± Dox and hMX3 ± Dox males and females at 6 mpf. Scale bars: 100 μm (**D**) ORO staining of frozen liver sections of hMX1 ± Dox and hMX3 ± Dox males and females at 4 mpf. Scale bars: 50 μm. (**E**) Molecular analysis of gMX2 and hMX3 NAFL revealed upregulation of selected lipid regulatory genes, including *ppar-γ, C/EBP-β, srebp1, fasn, acaca, chrebp, acc1,* and *dgat2*. (**F**) Biochemical analysis of triglycerides, total cholesterol, and oxidative stress was completed in triplicate in three four-month-old fish couples per group (WT ± Dox, hMX1 ± Dox, and hMX3 ± Dox). Statistically significant differences from the controls are indicated by * (*p* < 0.05), and ** (*p* < 0.01).

Whole-mount ORO staining indicated lipid deposits in the larval livers of hMXs + Dox (Figure 2B). Hence, the early fatty liver progressed in juvenile hMXs + Dox (3–4 mpf stages). As expected, the hMXs + Dox (hMX1 + Dox and hMX3 + Dox) groups at 24 dpf developed palpable liver steatosis (Figure 4A, panels 4 and 6), whereas control groups (Figure 4A, panels 1–3 and 5) showed no or few fat accumulations. H&E staining showed that hMXs + Dox hepatocytes suffered a ballooning degeneration progression (Figure 4A, panels 10 and 12), whereas such an effect was not observed in hepatocytes of the control groups (Figure 4A, panels 7–9 and 11). 

We next checked whether the early liver steatosis would develop to NAFLD in adult hMXs + Dox. Nearly 60% of the hMXs + Dox at 4 mpf started to display pale yellow livers compared with the control groups (Figure 4B). Furthermore, H&E staining analysis discovered that liver sections of hMXs + Dox adults displayed hepatocytes with various grades of lipid cytoplasmic vacuolation (Figure 4C; panels 2, 4, 6, and 8). On the other hand, regular liver sinusoids and hepatocytes displayed a vigorous cytoplasm and clear nucleus in control groups (Figure 4C; panels 1, 3, 5, and 7). Results of the H&E staining were verified by ORO staining, which apparently uncovered macrovesicular steatosis and massive lipid deposits in the hepatocytes of hMXs + Dox adults (Figure 4D; panels 2, 4, 6, and 8) compared with those in the control groups (Figure 4D; panels 1, 3, 5, and 7). hMXs + Dox adults displayed upregulated expressions of the selected liver lipogenic genes compared with that in the control groups (Figure 4E). As expected, the levels of hepatic triglycerides, cholesterol, and oxidative stress observed in hMXs + Dox were significantly greater than those in the controls (Figure 4F). These results indicate that Mxd3 overexpression can develop various grades of liver steatosis in the hMXs + Dox adults.

### 3.5. Mxd3 Overexpression Induces NASH Phenotypes in MXs+Dox Fish

**Figure 5:**
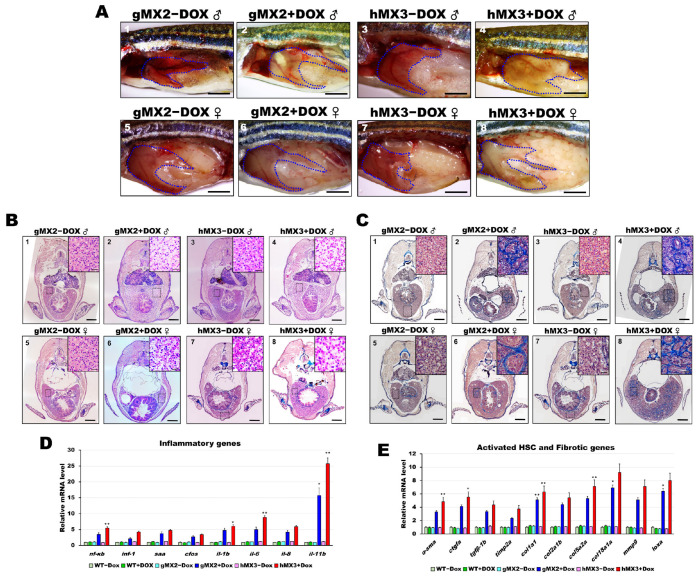
Nonalcoholic steatohepatitis (NASH) phenotypes in gMX2 and hM3 zebrafish at 8 mpf. (**A**) Gross liver of gMX2 ± Dox and hMX3 ± Dox male (panels 1–4) and female (panels 5–8) zebrafish at 8 mpf. The livers are circled. Scale bar: 2 mm. (**B**) H&E staining shown increased Mallory−Denk bodies (MDBs) with ballooned cells and lobular inflammation (panels 1–4 for male and panels 5–8 for female). Scale bar: 1 mm. Insets: normal hepatocytes (panels 1, 3, 5, and 7), MDBs and lobular inflammation (panels 2, 4, 6, and 8). Scale bar: 25 μm. (**C**) Masson’s trichrome staining of the liver tissue from gMX2 ± Dox and hMX3 ± Dox (panels 1–4 for male and panels 5–8 for female zebrafish). Scale bar: 1 mm. Insets: normal hepatocytes (panels 1, 3, 5, and 7); fibrosis (panels 2 and 6) and scar tissue linked with liver fibrosis (panel 4) and cirrhosis (panel 8). Scale bar: 25 μm. (**D**) qRT-PCR analysis of gMX2 and hMX3 NASH discovered upregulated inflammatory genes and (**E**) activated HSC and fibrotic genes. Statistically significant differences from the controls are indicated by * (*p* < 0.05), and ** (*p* < 0.01).

Previous studies had shown that NAFL may deteriorate the liver and induce NASH in zebrafish models [16,27,29,30,32,33,34,35]. Therefore, studies were conducted to examine whether Mxd3 is also important for developing NASH in the NAFLD of MXs + Dox. Over 55% of the 8 mpf gMX2+Dox and 65% of the 8 mpf hMX3 + Dox groups displayed pale yellow livers (Figure 5A). H&E staining increased Mallory−Denk bodies (MDBs) with ballooned cells and penetrated inflammatory cells, which are typical histopathological traits of NASH (Figure 5B; panels 2, 4, 6, and 8), and were more frequently (> 85%) seen in the male livers of the 8 mpf hMX3 + Dox (Figure 5B, panel 4) compared with those of controls (Figure 5B; panels 1, 3, 5, and 7). Masson’s trichrome staining also revealed increased numbers of MDBs formation of scar tissue in the hepatocytes of these groups concomitant with fibrosis and lobular inflammation than in hepatocytes of controls (Figure 5C).

As expected, the livers of gMX2 + Dox and hMX3 + Dox adults displayed upregulated expression of the selected inflammatory genes (Figure 5D) and fibrotic genes (Figure 5E) compared with their controls (Figure 5D). Taken together, these results show that hepatic Mxd3 overexpression gives rise to the progression of NASH phenotypes.

### 3.6. Chronic Effects of Hepatic Mxd3 Expression on NAHCC in Male MXs + Dox

**Figure 6:**
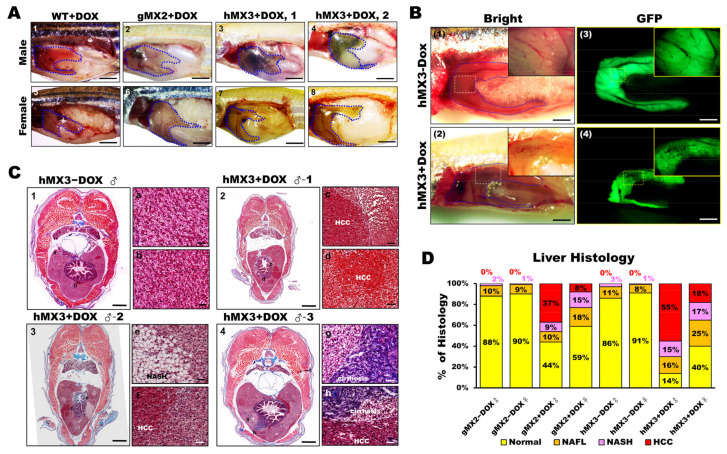
Male hMX3 zebrafish progress NAHCC at 10 mpf. (**A**) Gross liver of WT ± Dox, gMX2 ± Dox, and hMX3 ± Dox male (panels 1–4), and female (panels 5–8) zebrafish at 10 mpf. Scale bar: 3 mm. The livers are circled. Scale bar: 3 mm. (**B**) Angiogenesis and disruption of vascular architecture linked to HCC progression in the livers of hMX3 using bright field microscopy: (1) hMX3 − Dox and (2) hMX3 + Dox with corresponding GFP fluorescence images (3) hMX3 − Dox and (4) hMX3 + Dox. Scale bar: 3 mm. (**C**) Masson’s trichrome stained livers of male zebrafish (1) hMX3 − Dox indicative of normal liver, (2) hMX3 + Dox 1 (sample 1), (3) hMX3 + Dox 2, and (4) hMX3 + Dox 3. Scale bar: 2 mm. Insets: images of normal liver (panels a and b), HCC (panels c and d), steatohepatitis concomitant with HCC (panels e and f), and hMX3 + Dox 3 advanced fibrosis (cirrhosis) concomitant with HCC (panels g and h). Scale bar: 50 μm. (**D**) Percentage of histological features of liver sections from different experimental groups (*n* = 5−10). Masson’s trichrome staining results are shown as the percentages of fish displaying normal characteristics (yellow), steatosis (orange), NASH (pink), and HCC (red).

As hMX3 + Dox fish showed marked activation via inflammation (or oncogenesis) and accelerated NASH, we analyzed whether the livers of hMX3+Dox group were inclined to cancer progression from the beginning. By observing the MXs at 10 mpf, development of HCC was seen to occur earlier than 9 mpf in a fraction of male fish of hMX3s (1 and 2) + Dox (Figure 6A, panels 3 and 4). Distinct NASH phenotypes (such as steatosis, hepatitis/lymphocytic infiltration, portal fibrosis, and cholestasis) were seen in both males and females of gMX2 + Dox (Figure 6A, panels 2 and 6), as well as females of hMX3s (1 & 2) + Dox (Figure 6A, panels 7 and 8) than in WT controls (Figure 6A, panels 1 and 5). Furthermore, main hepatic vessels were seen in both hMX3 ± Dox (Figure 6B, panels 1–4), while only reticular veins were witnessed in the hMX3+Dox liver as a result of hepatic angiogenesis (Figure 6B; panels 2 and 4, insets). Masson’s trichrome stain revealed that clear HCC (Figure 6C, panel 2), steatohepatitis (Figure 6C, panel 3), and fibrosis concomitant with HCC (NAHCC) (Figure 6C, panel 4), and cirrhosis concomitant with HCC (Figure 6C, panel 4) was observed in male hMX3s (1–3) +Dox livers than in hMX3 − Dox (Figure 6C, panel 1). 

Furthermore, 60% male and 20% female hMX3 + Dox fish showed evidence of NAHCC. Quantification of histological inspection of the livers exposed that all the MXs + Dox had developed varying grades of HCC (from 8% to 55%). A total of 55% male hMX3 + Dox and 37% male gMX2 + Dox fish had advanced HCC, only 18% hMX3 + Dox and 8% gMX3 + Dox females had clear HCC, and the rest had NAFL or NASH compared with MXs − Dox fish (Figure 6D). Therefore, there was a dominant NAHCC development during HCC progression in male hMXs than in female hMXs.

### 3.7. Mxd3 Enhances Androgen Receptor (AR) Expression of NAHCC Progression in hMXs + Dox 

**Figure 7:**
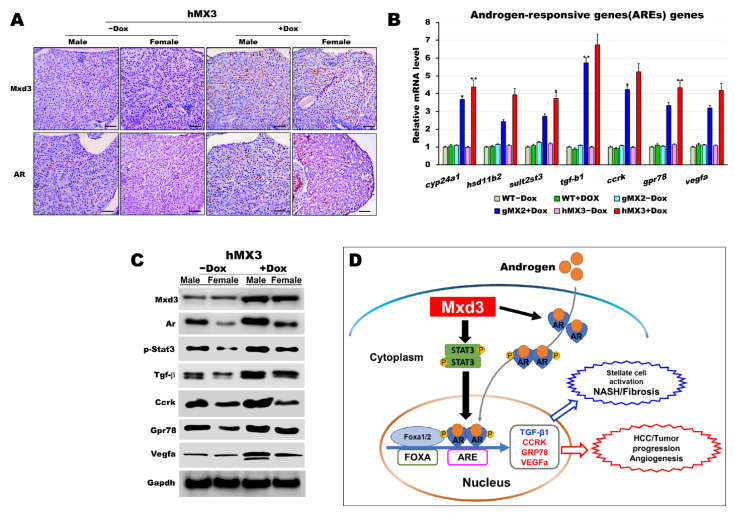
AR is certainly correlated with Mxd3 in the male hMX3. (**A**) AR and Mxd3 staining in the liver tissue of hMX3 ± Dox as determined by immunohistochemical analysis. Scale bar: 100 μm. (**B**,**C**) Molecular analysis of androgen-responsive gene expression by (**B**) qRT-PCR and (**C**) western blot analysis in the male livers of WT ± Dox, gMX2 ± Dox and hMX3 ± Dox. (**D**) Schematic diagram of the mechanism by which Mxd3 joins the NAHCC progression of the male hMX3 + Dox zebrafish. Mxd3 mediates the androgen receptor signaling in HCC. Statistically significant differences from the controls are indicated by * (*p* < 0.05), and ** (*p* < 0.01).

There is evidence with respect to the expression of AR in HCC [40,41,42,43]. AR-dependent gender differences in liver cancers have also been studied in mice [44,45] and zebrafish [38,46]. Therefore, we sought to discover the connection between Mxd3 and AR in hMXs + Dox. Because the predominance of HCC in male fish was more pronounced in hMXs + Dox than in WT controls (Figure 6D), we proposed that zebrafish Mxd3 might be involved in a similar androgen-signaling pathway to accelerate hepatocarcinogenesis in male hMXs. To determine the possible biological functions underlying Mxd3-regulated AR expression, we investigated the influence of Mxd3 on the regulation of AR activities during NAHCC progression in hMXs + Dox. As shown in Figure 7, a positive indicator between Mxd3 and AR expression was seen in the immunohistochemical analysis; Mxd3 and AR protein levels in both the male and female hMX3+Dox fish were significantly higher than in both the sexes of hMX3 − Dox (Figure 7A). We also observed transcriptional upregulation of the selected androgen-responsive genes (AREs) involved in the AR/androgen-signaling pathway (Figure 7B).

Moreover, consistent with western blot analyses, we observed an affirmative correlation between Mxd3 and AR protein expression and the selected ARE proteins, accompanied by higher contents of AR protein in both the male and female hMX3 + Dox fish than in both the sexes of hMX3 − Dox (Figure 7C). Thus, Mxd3 overexpression upregulated the AR at both the mRNA and protein levels in hMX3 + Dox. These data suggest that Mxd3 is positively correlated with AR in the hMX3 + Dox and further confirm that Mxd3 plays a conspicuous role in AR-mediated NAHCC (Figure 7D).

### 3.8. Sex Hormone Treatment Affected AR-Mediated NAHCC in Male MX3 + Dox Fish 

It has been reported that sex hormones affect HCC progression in the zebrafish [38,39,46] and rat [47] models as well as in humans [48,49,50]. In the current study, it was witnessed that male hMX3+Dox fish developed significantly aggressive HCC than female HCC hMX3 + Dox and controls fish (Figure 6D) and that Mxd3 played an important role in AR-mediated NAHCC (Figure 7D).

**Figure 8:**
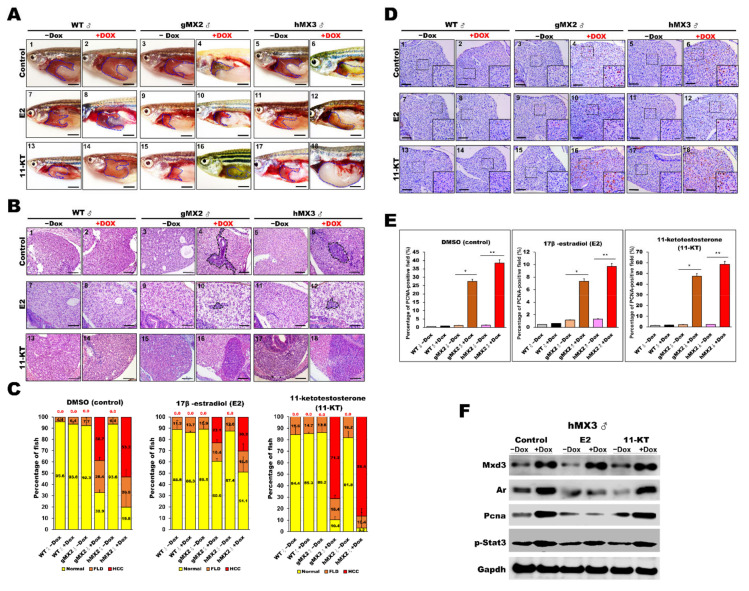
Treatment with sex hormones affects hepatocyte proliferation during NAHCC progression in adult gMX2 and hMX3 male fish. WT ± Dox, gMX2 ± Dox, and hMX3 ± Dox (6 mpf) groups were treated with E2 and 11-KT for 90 days. (**A**) Representative gross observations (Scale bar: 2.5 mm) and (**B**) liver histology of WT ± Dox, gMX2 ± Dox, and hMX3 ± Dox after no treatment (control) and treatment with E2 and 11-KT. Scale bar: 100 μm. (**C**) Percentage of NAHCC phenotypes observed from the liver histology in (**B**). Scale bar: 100 μm. (**D**) PCNA staining of liver slices of male zebrafish in distinct experimental groups. Scale bars, 100 μm. Insets, Scale bars, 25 μm. (**E**) Percentage of hepatocyte proliferation in the male zebrafish in discrete treatment groups (*n* = 5−10). (**F**) Western blot analysis in the different treatments of male hMX3 ± Dox livers. Statistically significant differences from the controls are indicated by * (*p* < 0.05), and ** (*p* < 0.01).

Hence, studies on treatment with sex hormones were carried out to evaluate their roles in NAHCC progression. Male MXs ± Dox and WT ± Dox adults were treated with either 17-estradiol (E2) or 11-ketotestosterone (11-KT). Representative gross observations of normal liver and HCC samples are presented in Figure 8A. After sex hormone treatment, the relative tumor sizes of male MXs adult (Figure 8A, panels 4 and 6) showed significant decreases with E2 treatment (Figure 8A, panels 10 and 12) compared with those of vehicle controls (Figure 8A, panels 1–3 and 5). As expected, the tumor sizes significantly increased only in the MXs + Dox after 11-KT treatment (Figure 8A, panels 16 and 18) compared with those of vehicle controls (Figure 8A, panels 13–15 and 17). Meanwhile, H&E staining also revealed similarly decreased tumor sizes in male MXs + Dox fish after E2 treatment and increased tumor sizes in these fish after 11-KT treatment (Figure 8B). A total of 38.7% male gMX2 + Dox and 53.3% male hMX2 + Dox fish had advanced HCC with control treatment, only 23.1% gMX2 + Dox and 30.3% hMX2 + Dox males had clear HCC with E2 treatment, the dramatic growth of HCC population in gMX2 + Dox (71.2%) and hMX2 + Dox (86.4%) after 11-KT treatment, and the rest had fatty liver disease (FLD) compared with MXs−Dox fish (Figure 8C).

PCNA staining was also carried out to survey the hepatocyte proliferation in MXs after sex hormone treatments (Figure 8D,E). After E2 treatment, the PCNA-positive staining reduced markedly in male MXs + Dox, indicating restrained cell growth in the liver tumors by the E2 treatment. After 11-KT treatment, the PCNA-positive cells increased significantly in male MXs + Dox, indicating that 11-KT boosted tumor growth. However, in vehicle control fish, both E2 and 11-KT showed no effect or had much less effect on cell proliferation. The protein expression level of PCNA showed a significant change in male hMX3+Dox livers with E2 or 11-KT treatment, which indicates a positive correlation with AR signaling (Figure 8F). Overall, the above assessments reveal that the E2 treatment decelerated whereas 11-KT treatment enhanced the HCC progression in male MX3 + Dox.

## 4. Discussion

We analyzed the MXD3 expression of the MYC/MAX/MAD network during adipogenic differentiation and the process of NAFLD in zebrafish. We discovered that, although MXD3 expression was initially linked to both lipogenesis and adipogenesis (Figure 2), it significantly induced adiposity and NAFLD (Figure 2 and Figure 4) during zebrafish development at juvenile (< 30 dpf), and young adult (< 4 mpf) stages. Most notable was the MXD3 overexpression, which induced strong expression of AR signaling (Figure 7) and dominant NAHCC development during HCC formation in male rather than female MXs (Figure 6). Our findings confirm and extend findings from previous studies that describe the expression of *mxd3* network genes linked to lipogenesis and carcinogenesis in NAHCC.

The expression levels of MXD3 were decreased three-fold in adult skeletal muscle tissues than in such tissues in the fetal period in Qinchuan cattle [51]. Depletion of *mxd3* led to a decrease in cell numbers, indicating that MXD3 is necessitated by cell proliferation [11,52]. By contrast, MXD3 overexpression is adequate to stimulate proliferation in mouse granule neuron precursors(GNPs) [52]. Surprisingly, MXD3 overexpression negatively regulated differentiation of mouse B cells [53]. Constant MXD3 overexpression, however, in mouse GNPs and human medulloblastoma cells, resulted in restrained cell proliferation as a result of the activated apoptosis [11,52]. Shimada et al. revealed that MXD3 is a new regulatory gene for adipogenesis in obese people. MXD3 expression is markedly increased in visceral fat in obese people as well as in DIO zebrafish model [17]. In the current study, Mxd3 overexpression increased both early (Figure 2D) and mature adipocyte marker genes (Figure 3J). Mxd3 overexpression can increase the adipogenic and lipogenic function (Figure 2). These results agreed with those reported previously that MXD3 could disrupt the MAX/MYC heterodimer, and reduce MYC activation to promote adipogenesis [15,17]. Thus, adipose tissue hyperplasia of gMXs fish (Figure 3) mainly occurred due to lipogenesis and adipogenesis of preadipocytes into adipocytes.

Low levels of serum adiponectin have been revealed to connect with many cancers, including breast, prostate, kidney, pancreatic, gastric, and colon cancers [54,55]. Adiponectin deficient mice treated with choline-deficient L-amino acid-defined diet [56] or high fat diets acquired liver cirrhosis and tumors [57]. Adipokines appear to have an imperative role, not only in the NAFLD progression, but also in NAHCC [58,59,60]. In our study, we found that MXD3 modulates adipokines in the adipose tissue of gMX2+Dox-treated zebrafish, and is accompanied primarily by obesity effects. The adiposity of gMX2+Dox correlated with increased expression of leptin and a decrease of adiponectin (Figure 3J). These results may have been due to oncometabolic stress related to NAFL (Figure 4) and NASH-related carcinogenesis in gMX2+Dox (Figure 5 and Figure 6).

Barisone et al., using an online bioinformatics platform to inquire cancer datasets, revealed that MXD3 is expressed in various cancers [61]. Xu et al. found that 10 hub genes, including MXD3, were aligned with HCC progression based on a gene co-expression network analysis [14]. Moreover, Ngo et al. also found that, across the datasets, MXD3 was highly overexpressed in HCC with a 2.88-fold change in relation to normal tissues [10]. The MXD3 promoter sequence region has a tendency to be hypomethylated in liver cancer in respect to adjacent tissue samples [10]. In several other studies across several models, it has been revealed that both depletion and MXD3 overexpression causes reduced cell proliferation [11,12,61]. These data suggest that it is important for MXD3 to maintain its cellular concentrations to function optimally. In this study, we discovered the possibility of upregulation of the *mxd3* through transgenic overexpression of *mxd3* in zebrafish and its liver. Specifically, Mxd3 overexpression leads to the progression of NAFLD phenotypes, including NASH, fibrosis, and HCC (Figure 6 and Figure 7).

HCC mainly influences the males, with a higher prevalence in male participants than in females [62,63]. The causes for the gender differences are complex in metabolic cytokines, such as adipokines and hepatokines that may cooperate with each other for maintaining liver health [64,65]. The zebrafish liver cancer also has statistically significant sexual dimorphism with male dominance; this has been confirmed by comparison of the male and female *kras^V12^* [38,46] and *Myc/xmrk* transgenic zebrafish [39]. In this study, we showed a similar sexual dimorphism in the liver cancer progression of hMX3 (Figure 6). Specifically, male MXs+Dox transgenic zebrafish developed significantly severe HCC than female MXs+Dox transgenic zebrafish (Figure 6D). We should note that the AR activation is aligned with human malignancy, such as prostate cancer, HCC, and pancreatic cancer, and that AREs, such as TGF-beta, CCRK, GRP78, and VEGF, play indispensable roles in the AR-mediated carcinogenesis [66,67,68]. Li et al. specifically depleted the *ar* gene in the liver of *kras^V12^* transgenic zebrafish and observed alleviated liver tumor progression in these zebrafish [38]. In agreement with these findings, we found that overexpression of MXD3 enhances AR expression and is accompanied by an increase in the AR-signaling pathways, resulting in hepatocarcinogenesis. (Figure 7C,D). In line with these results, we showed that sex hormones show important roles in HCC development. In particular, E2 could retard HCC (also NASH and cirrhosis) progression, particularly in both the male gMXs + Dox zebrafish (Figure 8A,B), while 11-KT could promote HCC progression predominantly in male hMX3+Dox zebrafish (Figure 8A–C). Additionally, cell proliferation in gMXs + Dox increased notably after 11-KT treatment and decreased markedly after E2 treatment, compared with those in the control groups (Figure 8D, E). In line with our findings, the response level of ARE proteins was accompanied by sex hormone treatment (Figure 8F). Generally, our results suggest that sex hormones affect NAHCC progression in the MXs zebrafish.

## 5. Conclusions

We showed that MXD3 is one of the factors that contributes towards an increase in visceral fat via the proliferation of preadipocytes and early adipogenesis. Overexpression of *mxd3* caused somatic growth and obesity, which then deteriorated the systemic lipid metabolic dysfunction (Mxd3 modulated adipokines in the adipose tissue of gMX2), ultimately resulting in dyslipidemia, liver steatosis, and NASH in MXs+Dox-treated zebrafish. Our results further show that *mxd3* expression increases both adipogenesis and carcinogenesis in AR-signaling associated NAHCC, indicating that the function of MXD3 on HCC growth is most closely linked to oncogenic AR signaling in the zebrafish liver. Our results also indicate that MXD3 plays a significant role in the regulation of adipokines and enhancement of the AREs, and then oncogenic AR signaling to join in the NAHCC progression. Thus, the functions of MXD3 on the modulation of adipokines and regulation of AR signaling could provide a novel therapeutic strategy for HCC.

## Data Availability

The datasets used in the current study are available from the corresponding author upon reasonable request.
